# Supplementary material for: CFPU: A Cell-Free Processing Unit for High-Throughput, Automated In Vitro Circuit Characterization in Steady-State Conditions
Source: Biodes Res. 2021 Mar 17;2021:2968181. doi: 10.34133/2021/2968181 (PMC10521719; doi:10.34133/2021/2968181)
Supplement: Supplementary Materials — Figure S1: characterization of PWM for on-chip mixing of lysate and energy solutions. Figure S2: disrupting steady-state expression. Figure S3: chip-to-chip reproducibility. Figure S4: GFP expression for a range of DNA dilutions. Figure S5: mixing 3 components on-chip with PWM. Figure S6: summary of CFPU experiments. Figure S7: overview of the LabView program. [file 2968181.f1.pdf]

Supplementary Figures

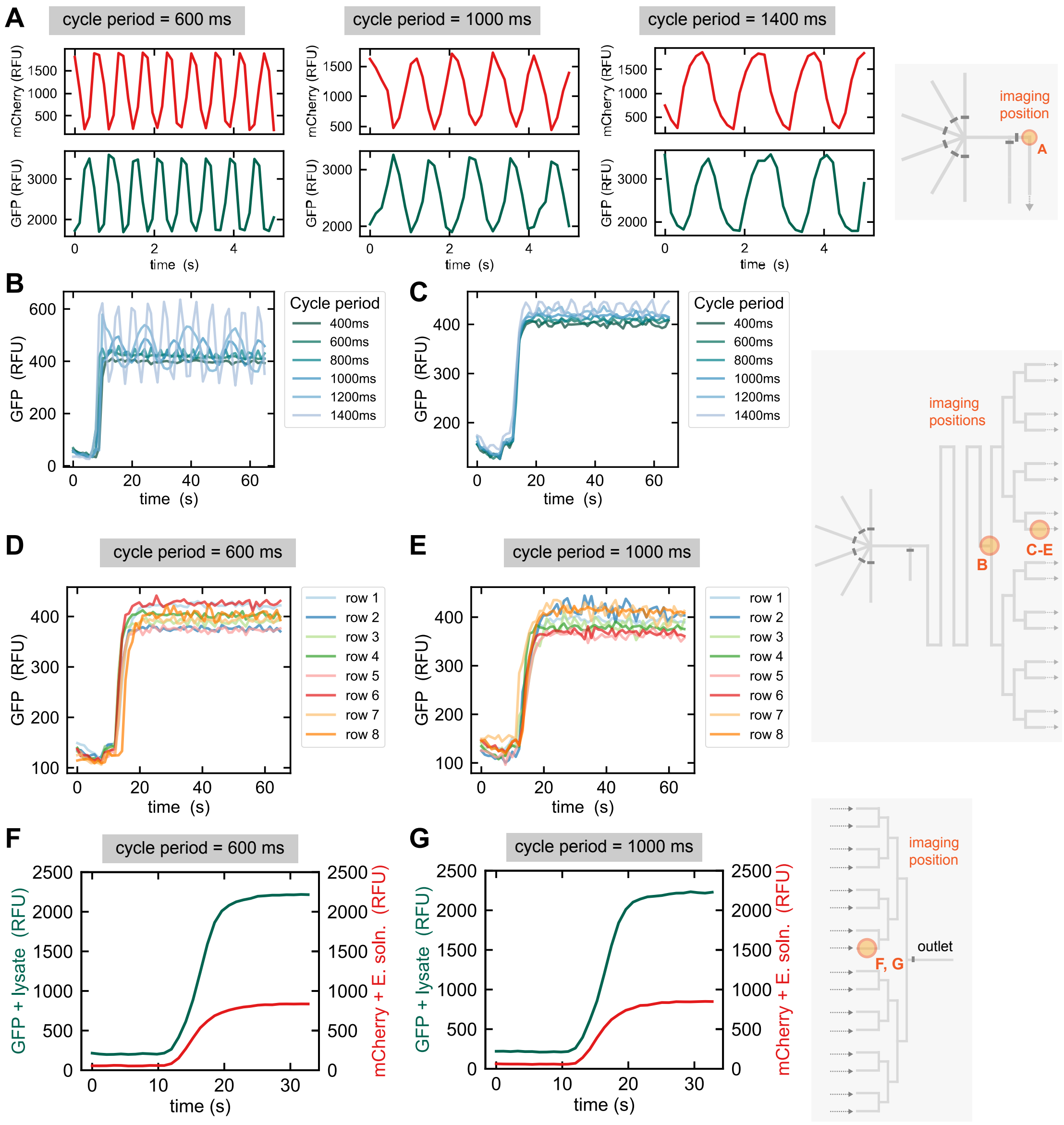

Figure S1: **Characterization of PWM for on-chip mixing of lysate and energy solutions.**

Caption on the next page.

Figure S1: **(A)** GFP (lysate) and mCherry (energy solution) signals versus time for cycle periods equal to 600, 1000 and 1400 ms. Images were acquired directly after the inlets, just before entering the serpentine channel. **(B, C)** GFP (lysate tracer) signal over time for a range of cycle periods as visualized at the end of the serpentine channel **(B)** and at the beginning of the exchange channel. **(D, E)** Time-lapse of the GFP (lysate tracer) signal acquired at the beginning of each exchange channel for cycle periods equal to 600 ms **(D)** and 1000 ms **(E)**. **(F, G)** GFP (lysate) and mCherry (energy solution) signals versus time for cycle periods equal to 600 ms **(F)** and 1000 ms **(G)** for images acquired at the end of the exchange channel. In all cases a duty cycle of 50% was maintained.

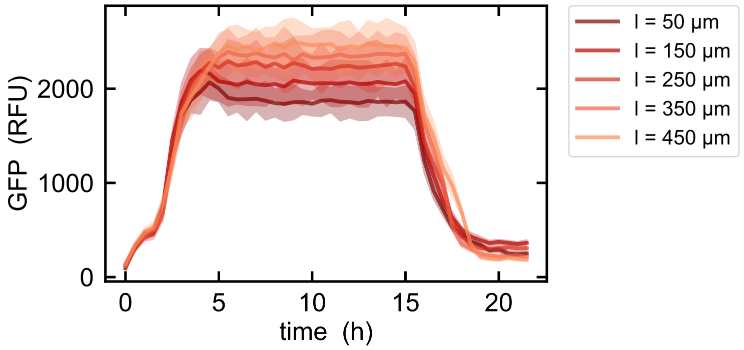

Figure S2: **Disrupting steady state expression.** Here we show the expression of GFP versus time for different unit cell connecting channel lengths. After 15 hours we stop flowing cell-free extract and instead flow PBS, showing that we can effectively terminate steady state GFP production in the absence of fresh reagents.

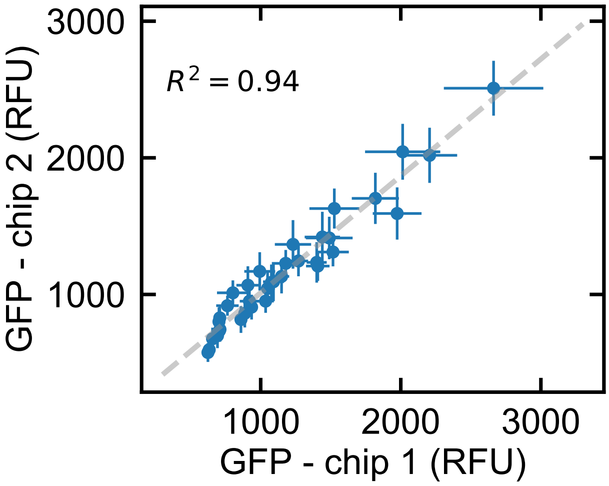

Figure S3: **Chip-to-chip reproducibility.** Steady state GFP expression values obtained for two separate chips for a range of DNA template dilutions. The same DNA microplate was used to spot both chips.

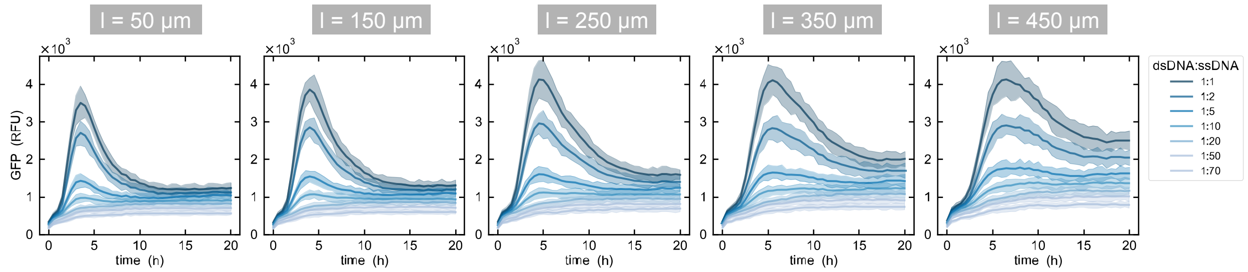

Figure S4: **GFP expression for a range of DNA dilutions.** GFP expression versus time for different ratios of dsDNA template to ssDNA oligo for each unit cell connecting channel length. All data presented represents mean values ± SD ($n = 8$)

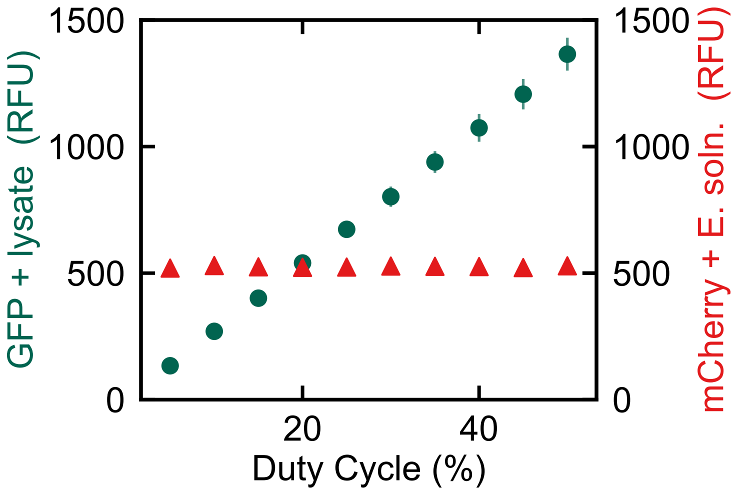

Figure S5: **Mixing 3 components on-chip with PWM.** Fluorescence intensity measured for GFP (lysate) and mCherry (energy solution) versus the duty cycle percentage corresponding to the lysate solution. The duty cycle for the energy solution was held constant at 50%.

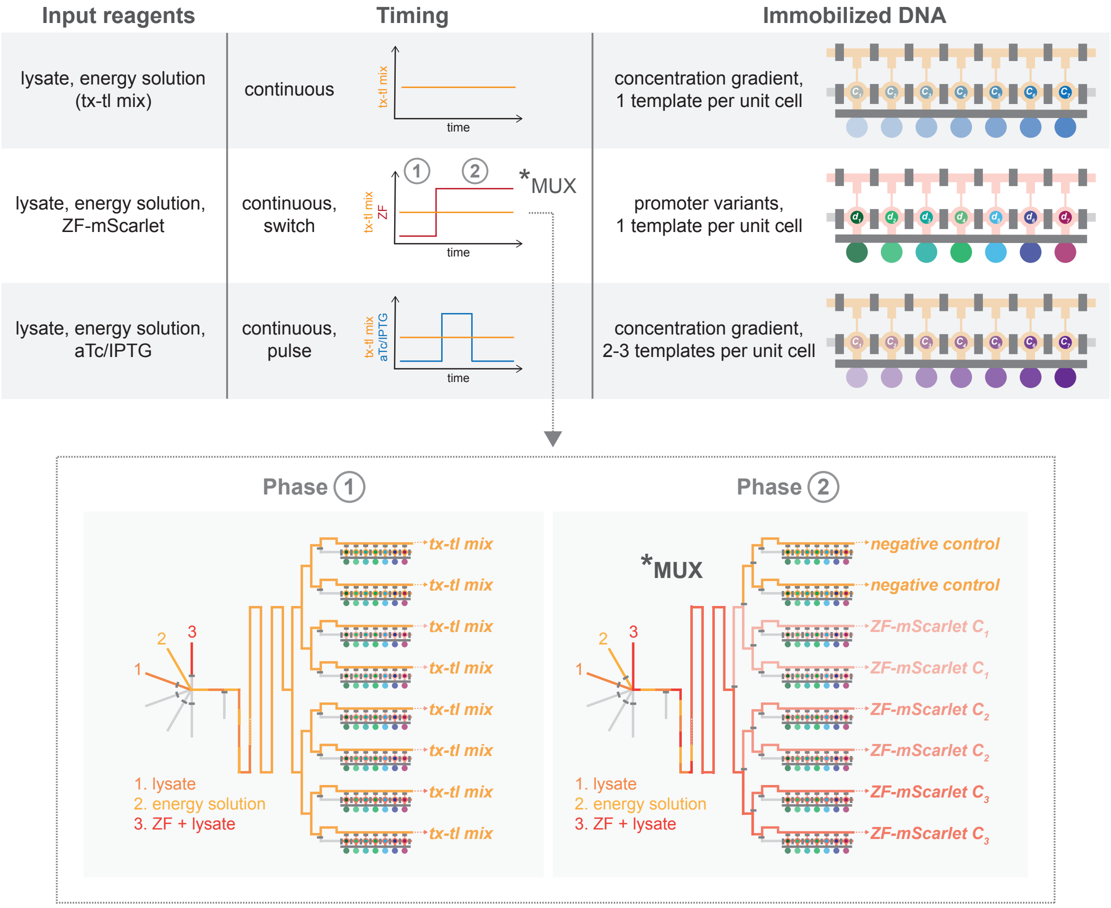

Figure S6: **Summary of CFPU experiments.** Three types of experiments were performed in this study corresponding with each section of the results: basic characterization, ZF combinatorics and small molecule perturbations and networks. The table highlights details of each experimental mode employed, including input reagents, timing and DNA templates. Different experimental phases and multiplexing is shown in the sketch below for the second experimental mode involving ZF combinatorics.

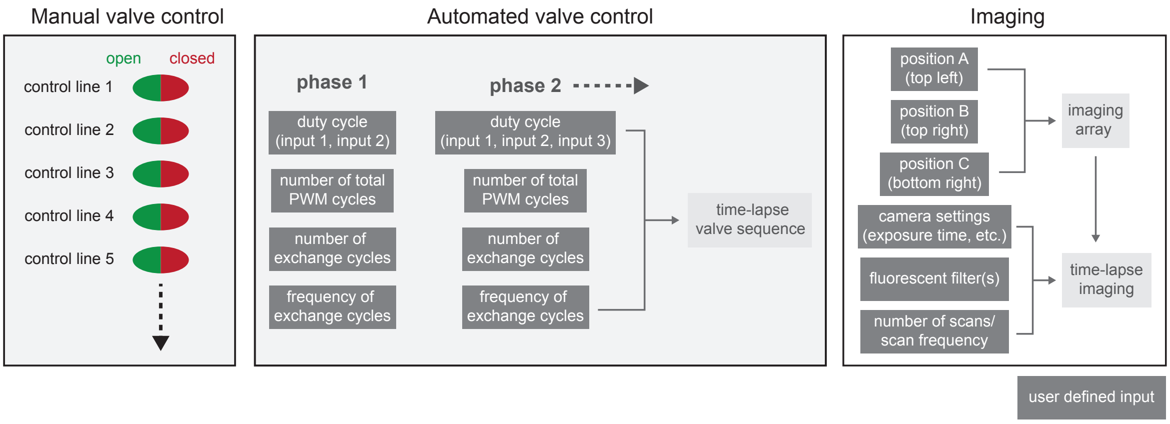

Figure S7: **Overview of the LabView program.** All control lines corresponding to the on-chip pneumatic valves can be toggled open or closed manually (left panel). This user interface is useful for priming the chip, as well as carrying out the surface chemistry and DNA immobilization. The pneumatic valves can also be controlled automatically over the course of an experiment (middle panel). The user defines key parameters corresponding with each phase of an experiment, and the program sequentially carries out each phase according to a hard-wired valve control sequence that specifies the inputs to mix and any multiplexing. While the experiment is running, imaging of the unit cells is automated based on user defined inputs (right panel). Saving three unit cell positions allows the program to define a scanning array that includes all unit cells to image at a designated frequency and according to other imaging parameters.
